# 1′-Methyl-2,2′′-dioxoindoline-3-spiro-2′-pyrrolidine-3′-spiro-3′′-indoline-4′,4′-di­carbonitrile

**DOI:** 10.1107/S1600536809028025

**Published:** 2009-07-22

**Authors:** P. Ramesh, S. S. Sundaresan, N. Vidhya Lakshmi, Paramasivan T. Perumal, M. N. Ponnuswamy

**Affiliations:** a Centre of Advanced Study in Crystallography and Biophysics, University of Madras, Guindy Campus, Chennai 600 025, India; b Organic Chemistry Division, Central Leather Research Institute, Adyar, Chennai 600 020, India

## Abstract

In the title compound, C_21_H_15_N_5_O_2_, the pyrrolidine ring adopts a twist conformation. Both the oxindole rings are planar [maximum deviations of 0.076 (1) and 0.029 (1) Å in the two rings] and are oriented at a dihedral angle of 72.7 (1)°. The crystal structure is stabilized by C—H⋯O, N—H⋯O, N—H⋯N and C—H⋯π inter­actions.

## Related literature

For the use of indole derivatives as bioactive drugs, see: Stevenson *et al.* (2000 ▶). They exibit anti-allergic, central nervous system depressant and muscle-relaxant properties, see: Harris & Uhle (1960 ▶); Ho *et al.* (1986 ▶). Indoles also exhibit high aldose reductase inhibitory activity, see: Rajeswaran *et al.* (1999 ▶). For puckering and asymmetry parameters, see: Cremer & Pople (1975 ▶); Nardelli (1983 ▶).
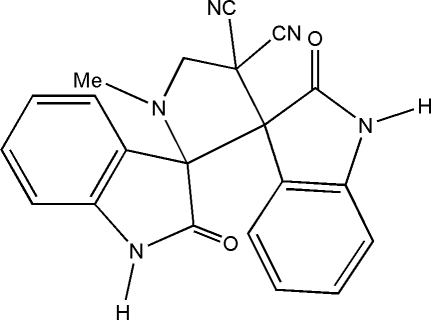


## Experimental

### 

#### Crystal data

C_21_H_15_N_5_O_2_
*M* *_r_* = 369.38
Monoclinic, 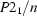

*a* = 13.3173 (3) Å
*b* = 9.9480 (2) Å
*c* = 13.3950 (3) Å
β = 91.827 (1)°
*V* = 1773.67 (7) Å^3^
*Z* = 4
Mo *K*α radiation
μ = 0.09 mm^−1^
*T* = 293 K
0.30 × 0.25 × 0.20 mm

#### Data collection

Bruker Kappa APEXII area-detector diffractometer
Absorption correction: multi-scan (*SADABS*; Sheldrick, 2001 ▶) *T*_min_ = 0.972, *T*_max_ = 0.982
26314 measured reflections
6939 independent reflections
4820 reflections with *I* > 2σ(*I*)
*R*_int_ = 0.030

#### Refinement

*R*[*F* ^2^ > 2σ(*F* ^2^)] = 0.050
*wR*(*F* ^2^) = 0.142
*S* = 1.02
6939 reflections
263 parameters
2 restraints
H atoms treated by a mixture of independent and constrained refinement
Δρ_max_ = 0.38 e Å^−3^
Δρ_min_ = −0.21 e Å^−3^

### 

Data collection: *APEX2* (Bruker, 2004 ▶); cell refinement: *SAINT* (Bruker, 2004 ▶); data reduction: *SAINT*; program(s) used to solve structure: *SHELXS97* (Sheldrick, 2008 ▶); program(s) used to refine structure: *SHELXL97* (Sheldrick, 2008 ▶); molecular graphics: *ORTEP-3* (Farrugia, 1997 ▶); software used to prepare material for publication: *SHELXL97* and *PLATON* (Spek, 2009 ▶).

## Supplementary Material

Crystal structure: contains datablocks global, I. DOI: 10.1107/S1600536809028025/bt2998sup1.cif

Structure factors: contains datablocks I. DOI: 10.1107/S1600536809028025/bt2998Isup2.hkl

Additional supplementary materials:  crystallographic information; 3D view; checkCIF report

## Figures and Tables

**Table 1:** Hydrogen-bond geometry (Å, °)

*D*—H⋯*A*	*D*—H	H⋯*A*	*D*⋯*A*	*D*—H⋯*A*
C5—H5⋯O2	0.93	2.49	3.1371 (15)	127
C17—H17⋯O1	0.93	2.47	3.1433 (16)	130
C20—H20*B*⋯O1	0.97	2.54	2.9986 (14)	109
C22—H22*B*⋯O2^i^	0.96	2.53	3.3713 (15)	147
N12—H12⋯O2^ii^	0.853 (17)	2.591 (17)	3.2114 (13)	130.5 (14)
N12—H12⋯N19^ii^	0.853 (17)	2.371 (17)	3.1613 (13)	154.4 (15)
C14—H14⋯O2^ii^	0.93	2.51	3.2305 (16)	135
N1—H1⋯N24^iii^	0.913 (18)	2.174 (18)	3.0315 (15)	156.1 (16)
C7—H7⋯*Cg*5^iv^	0.93	2.84	3.5366 (15)	156

Symmetry codes: (i) 
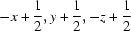
; (ii) 
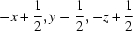
; (iii) 
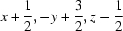
; (iv) 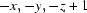. *Cg*5 is the centroid of the C13–C18 ring.

**Fig. 1.**
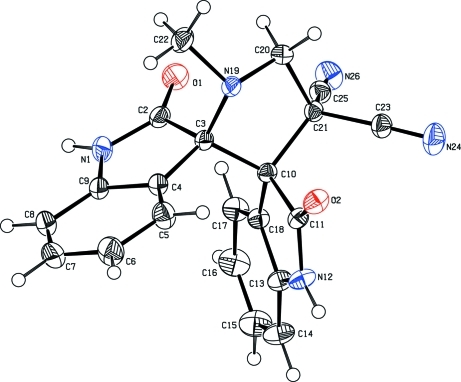
Perspective view of the molecule showing the displacement ellipsoids at the 50% probability level. The H atoms are shown as small circles of arbitrary radii.

**Fig. 2.**
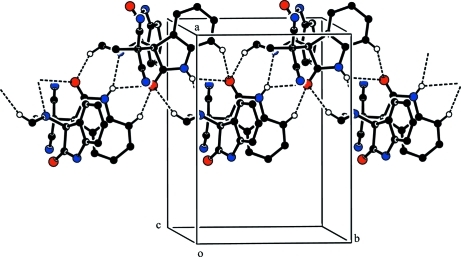
The crystal packing of the molecules viewed down c axis. H atoms not involved in hydrogen bonding have been omitted for clarity.

## supplementary crystallographic information

### Comment

Indole derivatives are used as bioactive drugs (Stevenson *et al.*, 2000) and they exibit anti-allergic, central nervous system depressant and muscle relaxant properties (Harris & Uhle 1960; Ho *et al.*, 1986). Indoles have been proved to display high aldose reductase inhibitory activity (Rajeswaran *et al.*,  1999).  Against this background and to ascertain the molecular conformation, the structure determination of the title compound has been carried out.

The ORTEP plot of the molecule is shown in Fig. 1. The pyrrolidine ring adopts a twist conformation with the puckering parameters (Cremer & Pople, 1975) and the asymmetry parameters (Nardelli, 1983) for this ring   q_2_ = 0.399 (1)Å, π = 202.5 (2)° and Δ2(C21) = 3.0 (1)°. Both   oxindole rings are planar and the keto atoms O1 and O3 deviate by 0.207 (1) and -0.093 (1)Å, respectively. The oxindole rings attached at 4 and 5 positions of the pyrrolidine ring are oriented at an angle of 72.7 (1)°. In the indole ring system, the endocyclic angles at C8 and C14 are contracted to 117.3 (1)° and 117.4 (1)°, while those at C9 and C13 are expanded to 122.2 (1)° and 122.5 (1)°, respectively. The cyano groups are almost linear which can be seen from the bond angles of 176.4 (1)° (C21-C23-N24) and 175.2 (1)° (C21-C25-N26). The sum of the bond angles around the hetero nitrogen atom in the oxindole ring systems are N1 [360.0°] and  N12 [359.8°] and in accordance with the *sp^2^* hybridization. The sum of the bond angles at N19 (334.4°)  in the pyrrolidine ring system is in accordance with the *sp^3^* hybridization.

The packing of the molecules in the crystal structure is stabilized by C-H···O, N-H···O,  N-H···N and C-H···π interactions.  Atom C22 (x, y, z) donates a proton to O2 (1/2-x, 1/2+y, 1/2-z) and forms a   zig-zag chain running along the b-axis.  The intermolecular N1-H1···N24 hydrogen bond forms a one dimensional chain running along the ac diagonal axis. The indole ring interacts with the other indole moiety through an intermolecular C-H···π interaction involving atom C7, the separation between H7 and the centroid (Cg5) of the ring (C13/C14/C15/C16/C17/C18) being 2.666Å.

### Experimental

A mixture of isatin, sarcosine and isatylidene malononitrile in methanol and a catalytic amount of silica gel (100–200 mesh) was added and refluxed for about 15 minutes. The precipitated solid was filtered, dried and purified by column chromatography to afford the pure product in 87% yield. The purified compound was recrystallized from ethanol by using slow evaporation method.

### Refinement

H atoms bonded to N were freely refined while the other H atoms were positioned geometrically (C—H = 0.93–0.97 Å) and allowed to ride on their parent atoms, with 1.5*U*_eq_(C) for methyl H and 1.2*U*_eq_(C) for other H atoms. The components of the anisotropic displacement parameters of (C21-C23) and (C21-C25) in the direction of  the bond between them were restrained to be equal within an effective standard deviation of 0.001.

### Crystal data

**Table:**

C_21_H_15_N_5_O_2_	*F*(000) = 768
*M**_r_* = 369.38	*D*_x_ = 1.383 Mg m^−^^3^
Monoclinic, *P*2_1_/*n*	Mo *K*α radiation, λ = 0.71073 Å
Hall symbol: -P 2yn	Cell parameters from 3546 reflections
*a* = 13.3173 (3) Å	θ = 2.1–33.5°
*b* = 9.9480 (2) Å	µ = 0.09 mm^−^^1^
*c* = 13.3950 (3) Å	*T* = 293 K
β = 91.827 (1)°	Block, colourless
*V* = 1773.67 (7) Å^3^	0.30 × 0.25 × 0.20 mm
*Z* = 4	

### Data collection

**Table:**

Bruker Kappa APEXII area-detector diffractometer	6939 independent reflections
Radiation source: fine-focus sealed tube	4820 reflections with *I* > 2σ(*I*)
graphite	*R*_int_ = 0.030
ω and φ scans	θ_max_ = 33.5°, θ_min_ = 2.1°
Absorption correction: multi-scan (*SADABS*; Sheldrick, 2001)	*h* = −18→20
*T*_min_ = 0.972, *T*_max_ = 0.982	*k* = −15→14
26314 measured reflections	*l* = −20→19

### Refinement

**Table:**

Refinement on *F*^2^	Secondary atom site location: difference Fourier map
Least-squares matrix: full	Hydrogen site location: inferred from neighbouring sites
*R*[*F*^2^ > 2σ(*F*^2^)] = 0.050	H atoms treated by a mixture of independent and constrained refinement
*wR*(*F*^2^) = 0.142	*w* = 1/[σ^2^(*F*_o_^2^) + (0.0723*P*)^2^ + 0.242*P*] where *P* = (*F*_o_^2^ + 2*F*_c_^2^)/3
*S* = 1.02	(Δ/σ)_max_ = 0.012
6939 reflections	Δρ_max_ = 0.38 e Å^−^^3^
263 parameters	Δρ_min_ = −0.21 e Å^−^^3^
2 restraints	Extinction correction: *SHELXL97* (Sheldrick, 2008), Fc^*^=kFc[1+0.001xFc^2^λ^3^/sin(2θ)]^-1/4^
Primary atom site location: structure-invariant direct methods	Extinction coefficient: 0.0042 (12)

### Special details

**Table:**

Geometry. All e.s.d.'s (except the e.s.d. in the dihedral angle between two l.s. planes) are estimated using the full covariance matrix. The cell e.s.d.'s are taken into account individually in the estimation of e.s.d.'s in distances, angles and torsion angles; correlations between e.s.d.'s in cell parameters are only used when they are defined by crystal symmetry. An approximate (isotropic) treatment of cell e.s.d.'s is used for estimating e.s.d.'s involving l.s. planes.
Refinement. Refinement of *F*^2^ against ALL reflections. The weighted *R*-factor *wR* and goodness of fit *S* are based on *F*^2^, conventional *R*-factors *R* are based on *F*, with *F* set to zero for negative *F*^2^. The threshold expression of *F*^2^ > σ(*F*^2^) is used only for calculating *R*-factors(gt) *etc*. and is not relevant to the choice of reflections for refinement. *R*-factors based on *F*^2^ are statistically about twice as large as those based on *F*, and *R*- factors based on ALL data will be even larger.

### Fractional atomic coordinates and isotropic or equivalent isotropic displacement parameters (Å^2^)

**Table:**

	*x*	*y*	*z*	*U*_iso_*/*U*_eq_	
O1	0.62030 (6)	0.88197 (10)	0.24064 (7)	0.0387 (2)	
O2	0.24158 (6)	0.73812 (9)	0.25781 (7)	0.03117 (19)	
N1	0.59318 (8)	0.75699 (11)	0.09828 (8)	0.0329 (2)	
H1	0.6578 (14)	0.7468 (18)	0.0787 (14)	0.058 (5)*	
C2	0.56573 (8)	0.82292 (11)	0.18087 (8)	0.0270 (2)	
C3	0.44951 (7)	0.81058 (11)	0.18735 (7)	0.02235 (19)	
C4	0.42192 (8)	0.73937 (11)	0.09067 (8)	0.0254 (2)	
C5	0.33077 (10)	0.71016 (14)	0.04484 (9)	0.0352 (3)	
H5	0.2712	0.7316	0.0755	0.042*	
C6	0.32903 (11)	0.64796 (15)	−0.04818 (10)	0.0417 (3)	
H6	0.2679	0.6272	−0.0799	0.050*	
C7	0.41773 (12)	0.61699 (14)	−0.09353 (10)	0.0417 (3)	
H7	0.4153	0.5740	−0.1552	0.050*	
C8	0.50971 (11)	0.64808 (13)	−0.04977 (9)	0.0362 (3)	
H8	0.5692	0.6283	−0.0811	0.043*	
C9	0.51005 (9)	0.70967 (11)	0.04223 (8)	0.0281 (2)	
C10	0.42527 (7)	0.73565 (10)	0.28602 (7)	0.02080 (18)	
C11	0.31810 (8)	0.67524 (11)	0.27584 (8)	0.0236 (2)	
N12	0.32571 (7)	0.54152 (10)	0.29159 (8)	0.0301 (2)	
H12	0.2748 (13)	0.4894 (17)	0.2909 (12)	0.045 (4)*	
C13	0.42464 (8)	0.50270 (11)	0.31500 (8)	0.0273 (2)	
C14	0.45870 (11)	0.37590 (13)	0.33844 (11)	0.0386 (3)	
H14	0.4152	0.3028	0.3391	0.046*	
C15	0.55997 (12)	0.36121 (14)	0.36089 (11)	0.0436 (3)	
H15	0.5852	0.2764	0.3767	0.052*	
C16	0.62440 (10)	0.46957 (14)	0.36045 (11)	0.0405 (3)	
H16	0.6923	0.4570	0.3762	0.049*	
C17	0.58894 (8)	0.59738 (13)	0.33672 (9)	0.0316 (2)	
H17	0.6325	0.6705	0.3365	0.038*	
C18	0.48791 (8)	0.61382 (11)	0.31355 (8)	0.0236 (2)	
N19	0.39864 (7)	0.93947 (9)	0.19956 (6)	0.02403 (18)	
C20	0.41979 (9)	0.98552 (11)	0.30134 (8)	0.0266 (2)	
H20A	0.3668	1.0442	0.3236	0.032*	
H20B	0.4831	1.0336	0.3059	0.032*	
C21	0.42485 (8)	0.85489 (11)	0.36474 (8)	0.02283 (19)	
C22	0.42339 (11)	1.04057 (13)	0.12517 (10)	0.0376 (3)	
H22A	0.4939	1.0603	0.1303	0.056*	
H22B	0.3857	1.1210	0.1368	0.056*	
H22C	0.4068	1.0068	0.0596	0.056*	
C23	0.33963 (8)	0.84184 (12)	0.43191 (8)	0.0281 (2)	
N24	0.27708 (9)	0.82688 (14)	0.48603 (9)	0.0446 (3)	
C25	0.51485 (8)	0.85674 (11)	0.43181 (8)	0.0268 (2)	
N26	0.57990 (8)	0.86218 (12)	0.48812 (8)	0.0392 (3)	

### Atomic displacement parameters (Å^2^)

**Table:**

	*U*^11^	*U*^22^	*U*^33^	*U*^12^	*U*^13^	*U*^23^
O1	0.0260 (4)	0.0448 (5)	0.0452 (5)	−0.0086 (4)	0.0027 (4)	−0.0073 (4)
O2	0.0199 (3)	0.0330 (4)	0.0406 (5)	0.0004 (3)	0.0001 (3)	0.0051 (3)
N1	0.0244 (5)	0.0381 (6)	0.0370 (5)	−0.0003 (4)	0.0122 (4)	−0.0043 (4)
C2	0.0234 (5)	0.0257 (5)	0.0323 (5)	−0.0019 (4)	0.0072 (4)	0.0021 (4)
C3	0.0203 (4)	0.0223 (5)	0.0247 (4)	−0.0001 (4)	0.0050 (3)	0.0000 (4)
C4	0.0275 (5)	0.0251 (5)	0.0240 (5)	0.0005 (4)	0.0052 (4)	0.0001 (4)
C5	0.0318 (6)	0.0435 (7)	0.0303 (5)	−0.0005 (5)	0.0015 (5)	−0.0043 (5)
C6	0.0428 (7)	0.0509 (8)	0.0311 (6)	−0.0057 (6)	−0.0035 (5)	−0.0051 (5)
C7	0.0594 (9)	0.0378 (7)	0.0283 (6)	−0.0034 (6)	0.0073 (6)	−0.0066 (5)
C8	0.0450 (7)	0.0327 (6)	0.0318 (6)	0.0024 (5)	0.0144 (5)	−0.0035 (5)
C9	0.0316 (5)	0.0250 (5)	0.0281 (5)	0.0010 (4)	0.0092 (4)	0.0008 (4)
C10	0.0175 (4)	0.0207 (4)	0.0242 (4)	−0.0003 (3)	0.0021 (3)	0.0001 (3)
C11	0.0212 (4)	0.0254 (5)	0.0243 (4)	−0.0031 (4)	0.0011 (4)	0.0022 (4)
N12	0.0246 (4)	0.0247 (5)	0.0408 (5)	−0.0067 (4)	−0.0023 (4)	0.0056 (4)
C13	0.0278 (5)	0.0239 (5)	0.0301 (5)	−0.0005 (4)	−0.0021 (4)	0.0036 (4)
C14	0.0427 (7)	0.0243 (5)	0.0485 (7)	−0.0005 (5)	−0.0063 (6)	0.0087 (5)
C15	0.0482 (8)	0.0310 (6)	0.0509 (8)	0.0102 (6)	−0.0085 (6)	0.0090 (6)
C16	0.0329 (6)	0.0388 (7)	0.0490 (7)	0.0105 (5)	−0.0097 (5)	0.0019 (6)
C17	0.0245 (5)	0.0308 (6)	0.0394 (6)	0.0025 (4)	−0.0022 (4)	−0.0011 (5)
C18	0.0222 (4)	0.0225 (5)	0.0261 (5)	0.0011 (4)	−0.0002 (4)	0.0007 (4)
N19	0.0274 (4)	0.0206 (4)	0.0243 (4)	0.0015 (3)	0.0058 (3)	0.0027 (3)
C20	0.0305 (5)	0.0207 (5)	0.0286 (5)	−0.0005 (4)	0.0027 (4)	−0.0004 (4)
C21	0.0204 (4)	0.0247 (5)	0.0236 (4)	−0.0003 (4)	0.0035 (3)	−0.0013 (4)
C22	0.0465 (7)	0.0291 (6)	0.0381 (6)	0.0048 (5)	0.0153 (5)	0.0108 (5)
C23	0.0242 (5)	0.0324 (6)	0.0279 (5)	0.0002 (4)	0.0044 (4)	−0.0010 (4)
N24	0.0340 (6)	0.0591 (8)	0.0414 (6)	−0.0017 (5)	0.0139 (5)	−0.0006 (5)
C25	0.0247 (5)	0.0283 (5)	0.0275 (5)	−0.0002 (4)	0.0025 (4)	−0.0031 (4)
N26	0.0328 (5)	0.0475 (7)	0.0370 (5)	0.0005 (5)	−0.0050 (4)	−0.0064 (5)

### Geometric parameters (Å, °)

**Table:**

O1—C2	1.2154 (14)	N12—H12	0.853 (17)
O2—C11	1.2134 (13)	C13—C14	1.3735 (16)
N1—C2	1.3467 (15)	C13—C18	1.3904 (15)
N1—C9	1.3993 (16)	C14—C15	1.380 (2)
N1—H1	0.913 (18)	C14—H14	0.9300
C2—C3	1.5578 (15)	C15—C16	1.378 (2)
C3—N19	1.4618 (13)	C15—H15	0.9300
C3—C4	1.5111 (15)	C16—C17	1.3896 (18)
C3—C10	1.5601 (14)	C16—H16	0.9300
C4—C5	1.3738 (17)	C17—C18	1.3809 (15)
C4—C9	1.3908 (15)	C17—H17	0.9300
C5—C6	1.3908 (18)	N19—C20	1.4572 (14)
C5—H5	0.9300	N19—C22	1.4607 (14)
C6—C7	1.380 (2)	C20—C21	1.5527 (15)
C6—H6	0.9300	C20—H20A	0.9700
C7—C8	1.376 (2)	C20—H20B	0.9700
C7—H7	0.9300	C21—C25	1.4749 (15)
C8—C9	1.3760 (16)	C21—C23	1.4763 (15)
C8—H8	0.9300	C22—H22A	0.9600
C10—C18	1.5103 (14)	C22—H22B	0.9600
C10—C11	1.5505 (14)	C22—H22C	0.9600
C10—C21	1.5871 (14)	C23—N24	1.1314 (14)
C11—N12	1.3502 (14)	C25—N26	1.1317 (15)
N12—C13	1.3989 (15)		
			
C2—N1—C9	111.95 (10)	C14—C13—C18	122.51 (11)
C2—N1—H1	125.0 (12)	C14—C13—N12	127.35 (11)
C9—N1—H1	123.0 (12)	C18—C13—N12	110.14 (9)
O1—C2—N1	127.18 (11)	C13—C14—C15	117.41 (12)
O1—C2—C3	125.15 (10)	C13—C14—H14	121.3
N1—C2—C3	107.67 (10)	C15—C14—H14	121.3
N19—C3—C4	113.97 (9)	C16—C15—C14	121.37 (12)
N19—C3—C2	113.70 (9)	C16—C15—H15	119.3
C4—C3—C2	101.77 (8)	C14—C15—H15	119.3
N19—C3—C10	102.43 (7)	C15—C16—C17	120.64 (12)
C4—C3—C10	116.80 (9)	C15—C16—H16	119.7
C2—C3—C10	108.52 (8)	C17—C16—H16	119.7
C5—C4—C9	119.56 (10)	C18—C17—C16	118.77 (11)
C5—C4—C3	132.01 (10)	C18—C17—H17	120.6
C9—C4—C3	108.28 (10)	C16—C17—H17	120.6
C4—C5—C6	118.92 (11)	C17—C18—C13	119.29 (10)
C4—C5—H5	120.5	C17—C18—C10	132.57 (10)
C6—C5—H5	120.5	C13—C18—C10	108.13 (9)
C7—C6—C5	120.24 (13)	C20—N19—C22	112.38 (9)
C7—C6—H6	119.9	C20—N19—C3	107.70 (8)
C5—C6—H6	119.9	C22—N19—C3	114.34 (9)
C8—C7—C6	121.67 (12)	N19—C20—C21	104.61 (8)
C8—C7—H7	119.2	N19—C20—H20A	110.8
C6—C7—H7	119.2	C21—C20—H20A	110.8
C9—C8—C7	117.32 (12)	N19—C20—H20B	110.8
C9—C8—H8	121.3	C21—C20—H20B	110.8
C7—C8—H8	121.3	H20A—C20—H20B	108.9
C8—C9—C4	122.26 (12)	C25—C21—C23	104.79 (9)
C8—C9—N1	127.61 (11)	C25—C21—C20	110.06 (9)
C4—C9—N1	110.06 (10)	C23—C21—C20	112.71 (9)
C18—C10—C11	102.13 (8)	C25—C21—C10	113.20 (8)
C18—C10—C3	117.56 (8)	C23—C21—C10	111.00 (9)
C11—C10—C3	108.98 (8)	C20—C21—C10	105.26 (8)
C18—C10—C21	116.82 (8)	N19—C22—H22A	109.5
C11—C10—C21	108.99 (8)	N19—C22—H22B	109.5
C3—C10—C21	102.18 (8)	H22A—C22—H22B	109.5
O2—C11—N12	126.68 (10)	N19—C22—H22C	109.5
O2—C11—C10	125.63 (10)	H22A—C22—H22C	109.5
N12—C11—C10	107.69 (9)	H22B—C22—H22C	109.5
C11—N12—C13	111.86 (9)	N24—C23—C21	176.43 (13)
C11—N12—H12	122.8 (11)	N26—C25—C21	175.22 (12)
C13—N12—H12	125.2 (11)		
			
C9—N1—C2—O1	−175.23 (12)	C11—N12—C13—C18	−1.10 (14)
C9—N1—C2—C3	4.82 (13)	C18—C13—C14—C15	−0.1 (2)
O1—C2—C3—N19	52.14 (15)	N12—C13—C14—C15	−178.85 (13)
N1—C2—C3—N19	−127.91 (10)	C13—C14—C15—C16	0.4 (2)
O1—C2—C3—C4	175.14 (12)	C14—C15—C16—C17	−0.3 (2)
N1—C2—C3—C4	−4.91 (11)	C15—C16—C17—C18	0.0 (2)
O1—C2—C3—C10	−61.12 (14)	C16—C17—C18—C13	0.28 (18)
N1—C2—C3—C10	118.84 (10)	C16—C17—C18—C10	179.26 (12)
N19—C3—C4—C5	−49.34 (16)	C14—C13—C18—C17	−0.21 (18)
C2—C3—C4—C5	−172.16 (13)	N12—C13—C18—C17	178.72 (10)
C10—C3—C4—C5	69.89 (16)	C14—C13—C18—C10	−179.42 (11)
N19—C3—C4—C9	126.17 (10)	N12—C13—C18—C10	−0.49 (13)
C2—C3—C4—C9	3.36 (11)	C11—C10—C18—C17	−177.43 (12)
C10—C3—C4—C9	−114.60 (10)	C3—C10—C18—C17	63.40 (16)
C9—C4—C5—C6	1.73 (19)	C21—C10—C18—C17	−58.64 (16)
C3—C4—C5—C6	176.83 (12)	C11—C10—C18—C13	1.63 (11)
C4—C5—C6—C7	−0.3 (2)	C3—C10—C18—C13	−117.54 (10)
C5—C6—C7—C8	−1.1 (2)	C21—C10—C18—C13	120.42 (10)
C6—C7—C8—C9	1.0 (2)	C4—C3—N19—C20	171.14 (9)
C7—C8—C9—C4	0.43 (19)	C2—C3—N19—C20	−72.83 (10)
C7—C8—C9—N1	−176.24 (12)	C10—C3—N19—C20	44.04 (10)
C5—C4—C9—C8	−1.83 (18)	C4—C3—N19—C22	−63.21 (12)
C3—C4—C9—C8	−177.99 (11)	C2—C3—N19—C22	52.83 (13)
C5—C4—C9—N1	175.36 (11)	C10—C3—N19—C22	169.69 (9)
C3—C4—C9—N1	−0.80 (13)	C22—N19—C20—C21	−161.08 (9)
C2—N1—C9—C8	174.33 (12)	C3—N19—C20—C21	−34.26 (10)
C2—N1—C9—C4	−2.67 (14)	N19—C20—C21—C25	132.70 (9)
N19—C3—C10—C18	−163.79 (9)	N19—C20—C21—C23	−110.72 (10)
C4—C3—C10—C18	70.95 (12)	N19—C20—C21—C10	10.40 (10)
C2—C3—C10—C18	−43.27 (12)	C18—C10—C21—C25	24.19 (12)
N19—C3—C10—C11	80.73 (10)	C11—C10—C21—C25	139.22 (9)
C4—C3—C10—C11	−44.53 (12)	C3—C10—C21—C25	−105.56 (9)
C2—C3—C10—C11	−158.75 (8)	C18—C10—C21—C23	−93.33 (11)
N19—C3—C10—C21	−34.50 (9)	C11—C10—C21—C23	21.70 (12)
C4—C3—C10—C21	−159.76 (8)	C3—C10—C21—C23	136.92 (9)
C2—C3—C10—C21	86.02 (9)	C18—C10—C21—C20	144.44 (9)
C18—C10—C11—O2	177.12 (10)	C11—C10—C21—C20	−100.54 (9)
C3—C10—C11—O2	−57.82 (14)	C3—C10—C21—C20	14.69 (10)
C21—C10—C11—O2	52.93 (14)	C25—C21—C23—N24	−44 (2)
C18—C10—C11—N12	−2.28 (11)	C20—C21—C23—N24	−164 (2)
C3—C10—C11—N12	122.78 (9)	C10—C21—C23—N24	78 (2)
C21—C10—C11—N12	−126.47 (9)	C23—C21—C25—N26	−30.2 (16)
O2—C11—N12—C13	−177.24 (11)	C20—C21—C25—N26	91.2 (16)
C10—C11—N12—C13	2.15 (13)	C10—C21—C25—N26	−151.3 (15)
C11—N12—C13—C14	177.75 (12)		

### Hydrogen-bond geometry (Å, °)

**Table:**

*D*—H···*A*	*D*—H	H···*A*	*D*···*A*	*D*—H···*A*
C5—H5···O2	0.93	2.49	3.1371 (15)	127
C17—H17···O1	0.93	2.47	3.1433 (16)	130
C20—H20B···O1	0.97	2.54	2.9986 (14)	109
C22—H22B···O2^i^	0.96	2.53	3.3713 (15)	147
N12—H12···O2^ii^	0.853 (17)	2.591 (17)	3.2114 (13)	130.5 (14)
N12—H12···N19^ii^	0.853 (17)	2.371 (17)	3.1613 (13)	154.4 (15)
C14—H14···O2^ii^	0.93	2.51	3.2305 (16)	135
N1—H1···N24^iii^	0.913 (18)	2.174 (18)	3.0315 (15)	156.1 (16)
C7—H7···Cg5^iv^	0.93	2.84	3.5366 (15)	156

Symmetry codes:  (i) −*x*+1/2, *y*+1/2, −*z*+1/2; (ii) −*x*+1/2, *y*−1/2, −*z*+1/2; (iii) *x*+1/2, −*y*+3/2, *z*−1/2; (iv) −*x*, −*y*, −*z*+1.
